# Age-Related Retinal Layer Thickness Changes Measured by OCT in *APP^NL-F/NL-F^* Mice: Implications for Alzheimer’s Disease

**DOI:** 10.3390/ijms25158221

**Published:** 2024-07-27

**Authors:** Lidia Sánchez-Puebla, Rosa de Hoz, Elena Salobrar-García, Alberto Arias-Vázquez, María González-Jiménez, Ana I. Ramírez, José A. Fernández-Albarral, José A. Matamoros, Lorena Elvira-Hurtado, Takaomi C. Saido, Takashi Saito, Carmen Nieto Vaquero, María I. Cuartero, María A. Moro, Juan J. Salazar, Inés López-Cuenca, José M. Ramírez

**Affiliations:** 1 Ramon Castroviejo Institute for Ophthalmic Research, Complutense University of Madrid, 28040 Madrid, Spain; lidsan02@ucm.es (L.S.-P.); rdehoz@med.ucm.es (R.d.H.); elenasalobrar@med.ucm.es (E.S.-G.); albearia@ucm.es (A.A.-V.); mrgjimenez@ucm.es (M.G.-J.); airamirez@med.ucm.es (A.I.R.); joseaf08@ucm.es (J.A.F.-A.); jomatamo@ucm.es (J.A.M.); marelvir@ucm.es (L.E.-H.); jjsalazar@med.ucm.es (J.J.S.); 2 Health Research Institute of the Hospital Clínico San Carlos (IdISSC), 28040 Madrid, Spain; 3 Department of Immunology, Ophthalmology and ENT, School of Medicine, Complutense University of Madrid, 28040 Madrid, Spain; 4 Department of Immunology, Ophthalmology and ENT, Faculty of Optics and Optometry, Complutense University of Madrid, 28040 Madrid, Spain; 5 Brain Science Institute, RIKEN, Laboratory for Proteolytic Neuroscience, Wako 351-0198, Japan; takaomi.saido@riken.jp; 6 Institute of Brain Science, Faculty of Medical Sciences, Nagoya City University, Nagoya 467-8601, Japan; saito-t@med.nagoya-cu.ac.jp; 7 Neurovascular Pathophysiology, Cardiovascular Risk Factor and Brain Function Programme, Centro Nacional de Investigaciones Cardiovasculares (CNIC), 28029 Madrid, Spain; carmen.nieto@cnic.es (C.N.V.); mariaangeles.moro@cnic.es (M.A.M.); 8 Hospital 12 de Octubre Research Institute (i + 12), 28041 Madrid, Spain; maricuar@ucm.es; 9 University Institute for Research in Neurochemistry, Complutense University of Madrid (UCM), 28040 Madrid, Spain; 10 Department of Pharmacology and Toxicology, Faculty of Medicine, Complutense University of Madrid (UCM), 28040 Madrid, Spain

**Keywords:** Alzheimer’s disease, *APP^NL/F-NF/L^*, C57BL/6J, OCT-SD, retinal layer

## Abstract

In Alzheimer’s disease (AD), transgenic mouse models have established links between abnormalities in the retina and those in the brain. *APP^NL-F/NL-F^* is a murine, humanized AD model that replicates several pathological features observed in patients with AD. Research has focused on obtaining quantitative parameters from optical coherence tomography (OCT) in AD. The aim of this study was to analyze, in a transversal case-control study using manual retinal segmentation via SD-OCT, the changes occurring in the retinal layers of the *APP^NL/F-NF/L^* AD model in comparison to C57BL/6J mice (WT) at 6, 9, 12, 15, 17, and 20 months of age. The analysis focused on retinal thickness in RNFL-GCL, IPL, INL, OPL, and ONL based on the Early Treatment Diabetic Retinopathy Study (ETDRS) sectors. Both *APP^NL-F/NL-F^*-model and WT animals exhibited thickness changes at the time points studied. While WT showed significant changes in INL, OPL, and ONL, the AD model showed changes in all retinal layers analyzed. The *APP^NL-F/NL-F^* displayed significant thickness variations in the analyzed layers except for the IPL compared to related WT. These thickness changes closely resembled those found in humans during preclinical stages, as well as during mild and moderate AD stages, making this AD model behave more similarly to the disease in humans.

## 1. Introduction

Alzheimer’s disease (AD) is the most common cause of dementia in the world [1]. This disease is characterized by an abnormal metabolism and the elimination of beta-amyloid (Aβ) and tau proteins, resulting in the formation of Aβ plaques and neurofibrillary tangles in the central nervous system (CNS) [2,3]. It is known that the misfolded proteins associated with AD play a role in the onset of tauopathy and inflammatory responses, leading to extensive synaptic and neuronal brain loss [4,5].

The brain, a component of the CNS, is uniquely observed through the retina. The neuroretina exhibits a variety of molecular and cellular characteristics shared with the brain, such as the presence of neurons, glial cells, an interconnected vascular network, and the presence of a blood–retina barrier [6,7,8,9]. Several studies have shown that the retina also develops changes because of AD in both humans [10,11,12] and transgenic mouse models [13,14,15,16,17]. Research using transgenic mouse models has established links between abnormalities in the retina and those in the brain [18,19].

Among these models is the *APP^NL-F/NL-F^* AD mouse model, which is an amyloidogenic model. The elevated ratio of Aβ_42_ in the *APP^NL-F/NL-F^* model leads to the formation of pathological Aβ deposits in the cerebral cortex and hippocampus. Consequently, this initiates the infiltration of microglia and astrocytes surrounding Aβ plaques starting at 6 months of age [20]. Likewise, this model replicates several pathological features observed in patients with AD, suggesting its usefulness as a preclinical model to study the role of amyloidosis in neuroinflammation as part of this disease [21]. In addition, early retinal vascular and structural changes that precede cognitive alterations cause the *APP^NL-F/NL-F^* model to exhibit behavior more like human AD than other transgenic models. In this murine model of AD, from 6 to 20 months, thickness changes in retinal layers have been observed via optical coherence tomography (OCT), showing the thinning and thickening of the total retina [13], as well as the outer and inner retina. The thinning of the inner retinal layers at 6, 12, and 15 months of age could be due to neurodegenerative processes, and the thickening of the outer retinal layers, mainly at 6 and 17 months of age, could represent a neuroinflammatory process.

Retinal imaging has undergone a revolution due to the use of OCT [22,23,24], which has been refined over the last 20 years to achieve an increasing resolution and speed. Specifically, spectral-domain (SD) OCT provides high resolution (approximately 5 micrometers) [25]. Additionally, the utilization of SD-OCT for small animal studies has enabled the acquisition of retinal images resembling histological cross-sections of the retina in experimental murine models [26]. The high acquisition speed and scan averaging contribute to a substantial reduction in speckle noise in mouse imaging, facilitating automated segmentation to quantify individual layers of the mouse retina [27]. Several studies have focused on obtaining quantitative parameters from OCT by segmenting the retinal layer in neurodegenerative diseases [28,29,30,31].

To the best of our knowledge, there have been no studies in which the individualized segmentation of each of the retinal layers has been analyzed in the *APP^NL-F/NL-F^* AD mouse model. This model develops brain and retinal alterations early, at 6 months, before the development of cognitive alterations, so its history is more similar to that of the human AD than other transgenic models.

The aim of this study was first to analyze, in a transversal case-control study using retinal segmentation via SD-OCT, the changes occurring in different retinal layers of the AD *APP^NL/F-NF/L^* model at 6, 9, 12, 15, 17, and 20 months of age in comparison to C57BL/6J. Second, this study sought to assess the changes that occur over time in the different retinal sectors and layers of both the APP model and the wild type (WT).

## 2. Results

### 2.1. Temporal Study of the WT Mice

**Figure 1:**
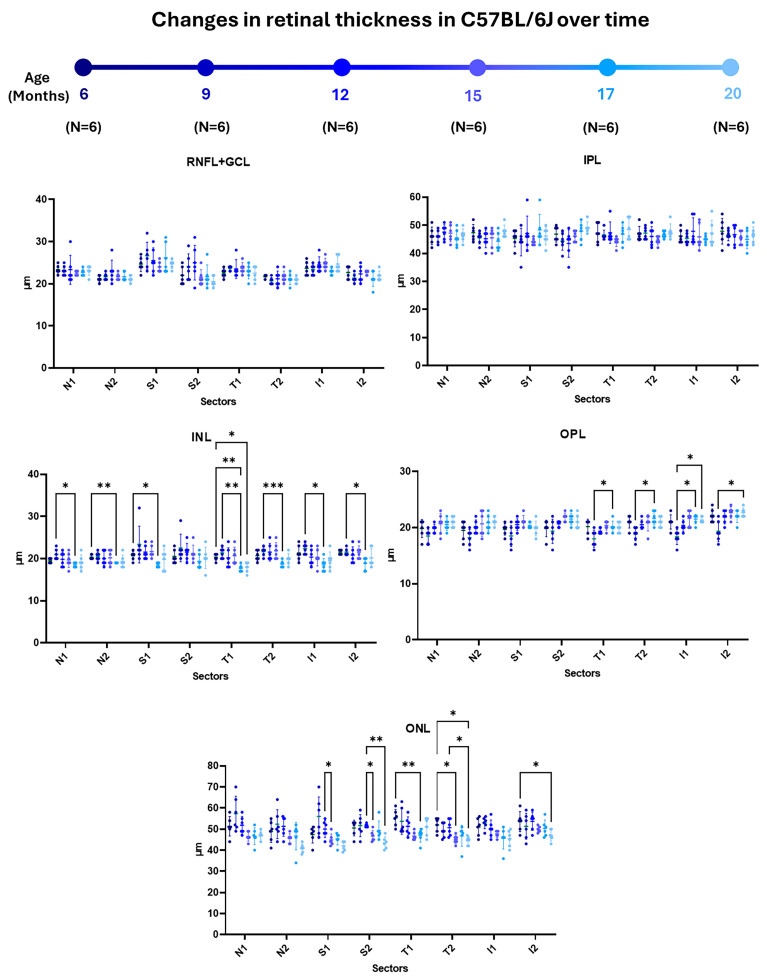
Representation of retinal-sector thickness in the different retinal layers of C57BL/6J mice at 6, 9, 12, 15, 17, and 20 months of age. For each time studied, n = 6. (RNFL-GCL: retinal nerve fiber layer and ganglion cell layer complex; IPL: inner plexiform layer; INL: inner nuclear layer; OPL: outer plexiform layer; ONL: outer nuclear layer. N1: inner nasal ring; N2: outer nasal ring; S1: inner superior ring; S2: outer superior ring; T1: inner temporal ring; T2: outer temporal ring; I1: inner inferior ring; and I2: outer inferior ring.) Significance values were set to * *p* < 0.05, ** *p* < 0.01, and *** *p* < 0.001.

When analyzing the **retinal nerve fiber layer, the ganglion cell layer complex (RNFL + GCL),** and the **inner plexiform layer (IPL)** of WT animals over time, we found no statistically significant changes to the thickness of these layers in any of the analyzed sectors (Figure 1 and Supplementary Table S1).

In the **inner nuclear layer (INL)**, there were significant decreases in most sectors between the initial stages (6 and 9 months) and in the more advanced stages of the study (17 months). Specifically, between the ages of 6 and 17 months, these decreases occurred in the N2, S1, and T1 sectors. There were also significant decreases between 9 and 17 months of age in the N1 sector and in both inferior rings (S1 and S2) (Figure 1 and Supplementary Table S1).

When the **outer plexiform layer (OPL)** was analyzed, there were significant increases only in the temporal and inferior sectors between the initial stages (6 and 9 months) and the later stages of the study time (17 and 20 months). Significant thickening was found between 9 and 17 months of the study in both T rings (T1 and T2) and the I1 sector. There was also a thickening in both the inferior rings (I1 and I2) between 9 and 20 months of age (Figure 1 and Supplementary Table S1).

In the **outer nuclear layer (ONL)**, there were significant decreases during the middle stages of the study in both the superior sectors and between the initial stages (6 months) and advanced stages (15, 17, and 20 months) in both the temporal sectors and in the inferior sector of the outer ring. In more detail, we also noted significant thinning between 12 and 15 months of age in both the superior sectors (S1 and S2). We also found thinning in the S2 sector between 12 and 20 months of the study. Between 6 and 17 months of age, we observed a significant decrease in the T1 sector, and between 6 and 15 months of age, we observed a significant decrease in the T2 sector. Between 6 and 20 months of the study, there was significant thinning in the T2 and I2 sectors, and there was also significant thinning between 12 and 20 months in the T2 sector only (Figure 1 and Supplementary Table S1).

### 2.2. Temporal Study of the APP^NL-F/NL-F^ Model

**Figure 2:**
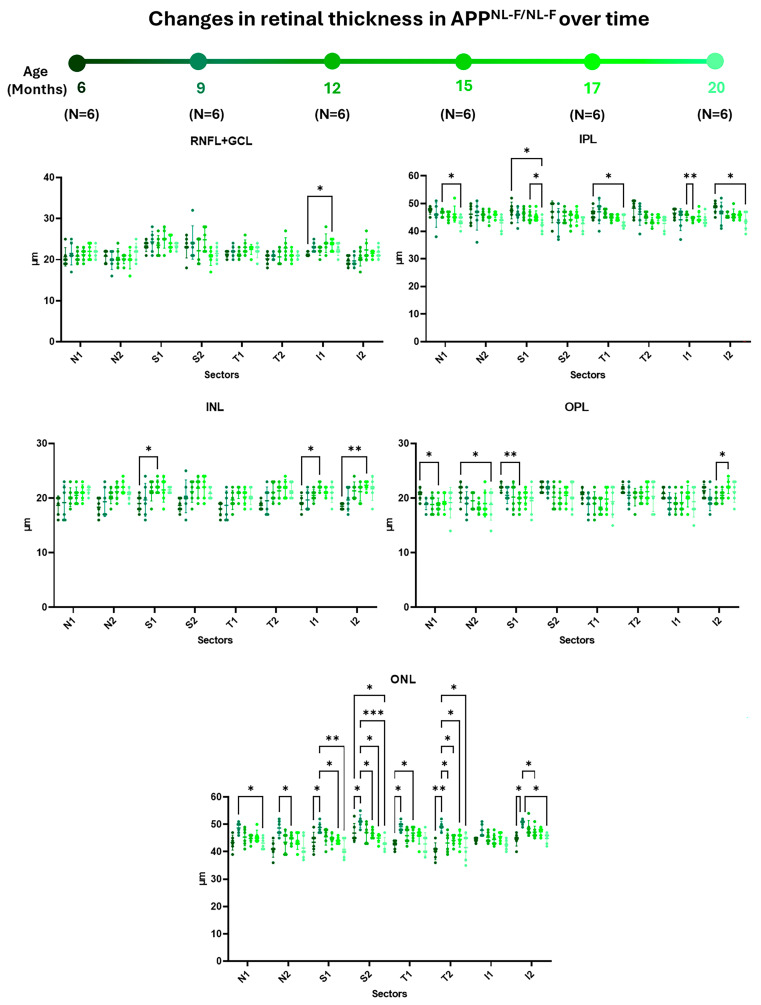
Representation of retinal-sector thickness in the different retinal layers of *APP^NL-F/NL-F^* mice at 6, 9, 12, 15, 17, and 20 months of age. For each time studied, n = 6. (RNFL-GCL: retinal nerve fiber layer and ganglion cell layer complex; IPL: inner plexiform layer; INL: inner nuclear layer; OPL: outer plexiform layer; ONL: outer nuclear layer. N1: inner nasal ring; N2: outer nasal ring; S1: inner superior ring; S2: outer superior ring; T1: inner temporal ring; T2: outer temporal ring; I1: inner inferior ring; and I2: outer inferior ring.) Significance values were set to * *p* < 0.05, ** *p* < 0.01, and *** *p* < 0.001.

When examining the **RNFL + GCL complex**, we observed a significant decrease in thickness only in sector I1 between 6 and 17 months of the study (Figure 2 and Supplementary Table S2).

In **IPL**, there was a statistically significant decrease in most of the analyzed sectors between the early stages (6 and 12 months) and the later stages (15 and 20 months) of the study. Specifically, we noted significant thinning between 6 and 20 months in the inner sectors S1, T1, and in I2. Moreover, we observed significant thinning in sector I1 between 12 and 15 months, in sector N1 between 12 and 20 months, and in sector S1 between 15 and 20 months in the study times (Figure 2 and Supplementary Table S2).

Within the **INL**, we observed thickening in certain sectors between the early stages (6 months) and the more advanced stages (15 and 17 months) of the study. Notably, there was statistically significant thickening between 6 and 15 months of age in sectors S1 and I1 and between 6 and 17 months in sector I2 (Figure 2 and Supplementary Table S2).

In the **OPL**, a thickness decrease was observed between the early stages (6 and 12 months) and the intermediate to late stages (15, 17, and 20 months) of the study. Specifically, between 6 and 15 months of age, there was significant thinning in retinal sectors N1 and S1. Additionally, thinning was observed in the N2 sector between 6 and 20 months of the study. Furthermore, there was thinning between 12 and 17 months of age in sector I2 (Figure 2 and Supplementary Table S2).

**In the ONL**, there was significant thickening in certain retinal sectors during the early stages (between 6 and 9 months) following thinning in most sectors between the early (9 months) and intermediate to late stages (12, 15, 17, and 20 months) of the study. Between 6 and 9 months of age, there was significant thickening in both superior and temporal sectors (S1, S2, T1, and T2), as well as in sector I2. This thickening was also evident in sector T1 between 6 and 15 months of the study. However, there was significant thinning between 9 and 15 months in all the external sectors (N2, S2, T2, and I2). Additionally, in this layer, between 9 and 17 months and between 9 and 20 months, significant thinning occurred in the superior sectors (S1 and S2), and it occurred only in sector S2 between 6 and 20 months. Additionally, between 9 and 20 months, significant thinning was observed in sector T2 and sector I2. Finally, in sector T2, thinning was noted between 9 and 17 months, as well as between 9 and 12 months of age (Figure 2 and Supplementary Table S2).

### 2.3. Comparative Study Comparing the WT and the APP^NL-F/NL-F^ at Different Time Points

#### 2.3.1. RNFL + GCL Complex

**Figure 3:**
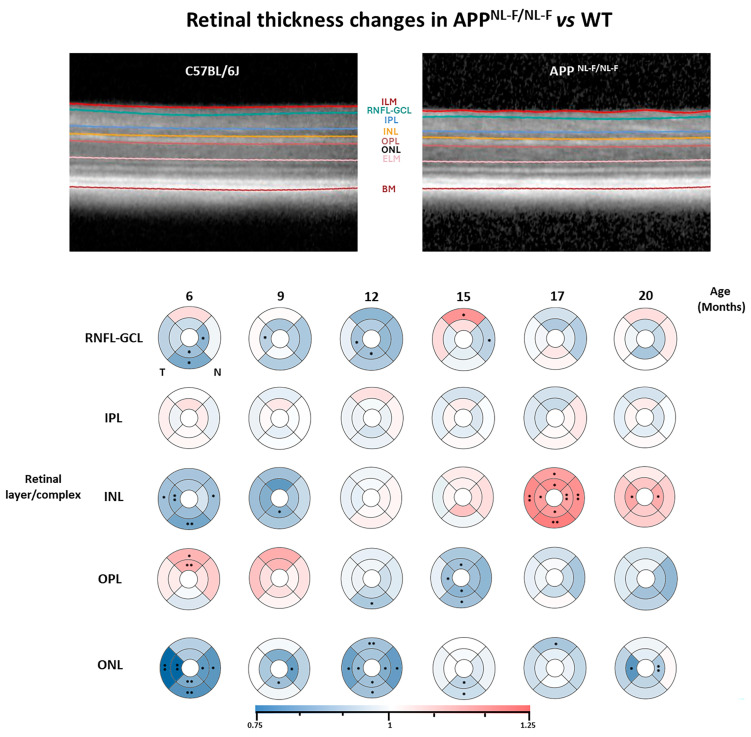
Representative images of the SD-OCT analysis and colorimetric representation of the retinal layers’ thickness differences in each study group between the *APP^NL-F/NL-F^* and WT groups. At each study time and in each group, n = 6. (RNFL-GCL: retinal nerve fiber layer and ganglion cell layer complex; IPL: inner plexiform layer; INL: inner nuclear layer; OPL: outer plexiform layer; and ONL: outer nuclear layer. ETDRS rings of 1-, 2-, and 3-mm diameters.) Blue tones: thinning; red tones: thickening. Significance values were set to • *p* < 0.05 and •• *p* < 0.01.

In this layer, when comparing the *APP^NL-F/NL-F^* model with the WT group, we mostly observed significant thinning throughout the analyzed times except at 15 months, when thickening was detected in the S2 sector. **At 6 months of age**, there was significant thinning in both inferior sectors and in sector N1 in the *APP^NL-F/NL-F^* model compared to the WT. **At 9 months of study**, only sector T1 showed significant thinning in the *APP^NL-F/NL-F^* model relative to the WT group. By **12 months of age**, the murine AD model exhibited significant thinning in sectors T1 and I1 compared to the WT. At **15 months of age**, the APP^NL-F/NLF^ model displayed significant thinning in sector N2, while sector S2 showed thickening compared to the WT group. No significant changes were observed during the late stages **(17 and 20 months)** in this layer between the study groups (Figure 3 and Supplementary Table S3).

#### 2.3.2. IPL

In this layer, there were no statistically significant changes observed at any analyzed time (Figure 2 and Supplementary Table S3).

#### 2.3.3. INL

In the **INL**, when we compared the *APP^NL-F/NL-F^* model versus the WT, significant thinning was observed in the early stages, while thickening was noted in the later stages. **At 6 months** in the *APP^NL-F/NL-F^* model, significant thinning occurred in sectors T1, T2, I2, and N2. **At 9 months**, thinning was observed in all sectors, though significantly only in sector I1. **At 12 and 15 months**, no significant changes were observed in any of the analyzed sectors. However, at **17 months of study,** there was a marked thickening that reached significance in all the retinal sectors analyzed (S1, S2, T1, T2, I1, I2, N1, and N2). Finally, at 20 months, generalized thickening was observed, which was significant only in sectors T1 and N1 (Figure 3 and Supplementary Table S3).

#### 2.3.4. OPL

In the **OPL**, for the *APP^NL-F/NL-F^* model, there was thickening in almost all sectors in the early stages, which shifted to thinning from 12 months onwards, and this thinning was significant at **12 and 15 months** compared to the WT. **At 6 months,** significant thickening was seen in both superior sectors (S1 and S2), persisting at 9 months without significance. **From 12 months**, significant thinning was observed in sector I2, which continued **at 15 months,** along with sectors S1, T1, I1, and I2. **At 17 and 20 months**, a trend towards thinning was noted in most sectors without reaching statistical significance (Figure 3 and Supplementary Table S3).

#### 2.3.5. ONL

In this layer, significant thinning was observed across all the study times in the *APP^NL-F/NL-F^* model compared to the control. **At 6 months**, significant thinning occurred in all sectors (T1, T2, I1, I2, N1, and N2) except for the superior ones. **At 9 months**, thinning was significant in sectors I1 and N1. **At 12 months**, significant thinning occurred in all sectors (S2, T1, T2, I1, I2, N1, and N2) except the S1 sector. **At 15 months**, thinning was significant in both the inferior sectors (I1 and I2). At **17 months**, only sector S2 reached significance. Finally, at **20 months**, significant thinning was observed in N1 and T1 compared to the WT group (Figure 3 and Supplementary Table S3).

## 3. Discussion

The main risk factor for developing AD is older age [32]. Therefore, the study of aging is highly important when analyzing this disease. Among the animal models most used to study human aging, mice are particularly suitable due to their short life expectancy. These models have contributed significantly to the understanding of the neurochemical and behavioral changes associated with aging [33]. Among WT mice, one of the most-used mice is the C57BL/6J strain, which was used in this study as a control group. In these mice, aging is characterized by a physical decline from 6 months of age and cognitive deterioration at 22 months; the relative age of this aging process occurs earlier than it does in humans [34].

On the other hand, among the models used for the study of AD, the *APP^NL-F/NL-F^* transgenic model is a second-generation model characterized by possessing two mutations: the *Swedish mutation (NL*) and the *Iberian mutation Beyreu-ther/Iberian (F)*. The first mutation increases the expression of Aβ_40_ and Aβ_42_, while the second overexpresses Aβ_42_ levels relative to Aβ_40_, leading to amyloidosis that is very similar to that observed in human AD. This model is characterized by the formation of Aβ plaques in the cerebral cortex and hippocampus, brain regions affected early in AD, triggering microglial and macroglial infiltrates around these plaques from 6 months of age [21,35].

In the present study, we analyzed a WT and AD model over a period ranging from 6 months to 20 months of age. In the C57BL/6J strain, thinning was observed in some retinal layers with aging, reaching significance only in certain sectors of the INL and ONL layers. These data are difficult to compare with those of other authors who have analyzed retinal-thickness changes in this strain, as their study periods were very early, ranging from 2 weeks to 2 months [36,37] and from 3 to 5 months [38]. Only one study analyzed this strain at a later time [39]. In that study, the authors analyzed the retinal thickness of this strain across four age groups (Group 1: 2–6 months; Group 2: 12–18 months; Group 3: 12–24 months; and Group 4: 24–32 months), finding significant reductions in the total retinal thickness when comparing Group 1 with the other groups. In the ONL, these significant thickness decreases also appeared, specifically when comparing Group 1 mice with the other groups and Group 2 with Group 4. In our work, consistent with the previously mentioned results, we also found statistically significant thinning in the ONL when comparing younger mice (6 months) with mice aged 15, 17, and 20 months. Thickness reductions were also observed when comparing 12-month-old mice with those aged 15 and 20 months. A possible explanation for this thickness reduction could be a loss of neuronal bodies and synapses [39]. This latter process would require microglial activation to perform synaptic stripping in the OPL [40,41] so that generalized thickening was observed in this layer instead of thinning.

When analyzing the *APP^NL-F/NL-F^* model at different time points, we observed statistically significant changes in some sectors across all analyzed layers. In the RNFL + GCL complex, there was a trend towards increased thickness over time. This thickness increase is similar to that found in the present study in the INL and at early time points in the ONL. The significant thickening observed in the RNFL + GCL complex was specifically found in the I1 sector between 6 and 17 months. These layers were studied as a complex due to the difficulty of distinguishing them in OCT images, as previously reported in other studies [42]. This thickness increase in the RNFL has also been reported in the 3xTg-AD model [43], in contrast to the findings of Song et al., who observed the thinning of this layer in the same transgenic model [44].

A thickness increase was also observed in the *APP^NL-F/NL-F^* model in the INL, reaching statistical significance between 6 and 15 months in the S1 and I1 sectors and between 6 and 17 months in the I2 sector. However, this was not reported for any of the murine models analyzed with OCT in AD [45]. In a previous study with the same animal model, it was shown that there was an increase in microglial activation due to a neuroinflammatory process that could explain the increase in thickness in this layer [13].

Lastly, in the *APP^NL-F/NL-^*^F^ model, we also observed thickening in the early stages of the disease in the ONL, which persisted at 9 months in most sectors of the ONL. These thickness increases could be due to a rise in glial activation, which has already been described in this model in the brain around Aβ plaques, specifically in the cortex and hippocampus, from 6 months of age [21]. However, at later time points in this same layer, we observed significant thinning between 9 and 15, as well as 17 and 20, months of age. In a model like *APP^NL-F/NL-F^*, but one that also incorporated the *Arctic mutation “G”*, the authors described this reduction in ONL thickness between 12 and 18 months of age [46].

In the humanized murine model analyzed in the current study, as the age of the mice advanced, we observed a trend towards thinning in the plexiform layers. In the IPL, this thinning reached statistical significance between the earliest study times (6 and 12 months) and the later times (15 and 20 months), and this thinning was more pronounced in the inner-ring sectors. Similar thinning trends were also observed in the OPL between the earlier months of the study (6 and 12 months) and the later times (15, 17, and 20 months), in the sectors of both the inner and outer rings. This decrease in the plexiform layers has been reported for the 3xTg-AD model in various previous studies at very early time points [47] and at times more like those studied in this work, considering these plexiform layers as part of the INL and GCL [43,48]. As these layers contain the synapses of most retinal neurons, the loss of thickness could correspond to secondary degeneration, a phenomenon in which the synapses and neurons near the originally affected neurons are also affected [48].

**Figure 4:**
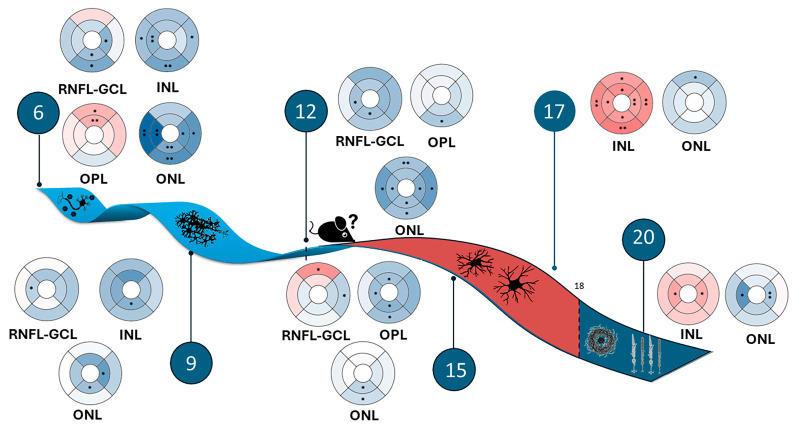
Illustrative diagram of the structural findings of the retina of the *APP^NL-F/NL-F^* murine model compared to the WT model. The timeline reveals early neurodegenerative processes caused by the accumulation of beta-amyloid oligomers, followed by neuroinflammatory processes mediated by the activation of macro- and microglia and, ultimately, the accumulation of beta-amyloid plaques, leading to the neurodegeneration of the outer retina and the death of photoreceptors. Blue tones: thinning; red tones: thickening. Significance values were set to • *p* < 0.05 and •• *p* < 0.01.

In the transversal case-control study comparing the *APP^NL-F/NL-F^* model with the WT group, we found that almost all study time points showed a statistically significant decrease in the thickness of the RNFL + GCL complex. This decrease in thickness is a typical feature of the disease, and it has been reported in studies conducted in patients with AD [49,50]. An illustrative diagram of the retinal structural findings of the APPNL-F/NL-F murine model compared to the WT model can be found in Figure 4.

In different murine models of AD, this thinning of the RNFL has also been observed, both at early times (at 4 months of age) in the TgCRN8 model [42] and at later times (6, 12, and 17 months) in the 5xFAD model [51], as well as at 15 and 16 months in the 3xTg-AD model [44], compared to controls. Histological studies have been conducted to explain this thickness decrease, demonstrating a loss in the ganglion cell number [42]. This decrease in RNFL thickness was reported in a previous study by our group, and it could be explained by the neurotoxic effects of soluble Aβ oligomers in the early stages of the disease [13]. For the 5xFAD model, the authors confirmed the decrease in RNFL + GCL thickness via the attenuation of pSTR in electroretinograms [51], another characteristic observed in patients with AD [52].

The thickness decrease was also significant in the INL at the earliest stages of the *APP^NL-F/NL-F^* model (6 and 12 months) compared to the control. Similarly, in the INL-OPL complex of 3xTg-AD mice, significantly lower thicknesses were observed at 4, 8, 12, and 16 months of age [43]. However, at 17 months in the *APP^NL-F/NL-F^* model, there was marked, significant thickening in this layer in all sectors, persisting until 20 months of age compared to the control. This thickening has not been reported in any previous study, and it could be explained by an inflammatory process limited only to this layer. This finding may have gone unnoticed by other authors because they did not study their models until such late times or due to the study of this layer within a complex or total retinal thickness.

When comparing the thickness of the OPL in the transgenic model with the WT group, we observed initial thickening. In AD, there is an inflammatory component that could occur from the preclinical stages of the disease [53]. On the other hand, this early thickening coincides with the statistically significant thinning of the INL at 6 months of age. This thickening aligns with that found by Batista et al. in the 3xTg-AD model [43]; however, they observed it throughout their study period (up to 16 months), whereas in our work, this initial thickening turned into significant thinning at 12 and 15 months of age. This thinning persisted, although no longer statistically significant, until 20 months of age, possibly as a compensatory mechanism for the significant thickening of the INL. This mechanism of compensation between neighboring layers was previously described by our research group [54].

In a previous study of patients with mild AD, statistically significant thinning was reported in the OPL when compared to healthy controls [11]. This finding would be consistent with those found at 15 months in the *APP^NL-F/NL-F^* murine model, corresponding to the age of 60 years in humans, when the murine model also shows a statistically significant thinning in this layer.

The changes found in this transgenic model in the INL and OPL resemble those found in early stages in subjects at high genetic risk for developing AD [55]. It is noteworthy that, at this early stage, the murine model does not exhibit behavioral changes; however, it does present biomarkers such as the presence of Aβ in the hippocampus and cerebral cortex [20], which would correspond in humans to a preclinical stage of AD.

Finally, in the ONL, we found statistically significant thinning in different sectors at all time points studied; it was more widespread at 6 and 12 months. These findings are consistent with those found in the *APP^NLF-G^* model, in which the authors also reported this significant thinning from 12 to 18 months in this same layer [46]. In the 3xTg-AD model, the results regarding this layer are controversial. While one study reported the thinning of this layer between 12 and 16 months [43], another study demonstrated statistically significant thickening from 4 to 16 months [48]. These discrepancies within the same model could be due to authors using different analysis protocols in each study, as well as different software/algorithms for retinal-layer segmentation.

Our findings have several implications: To the best of our knowledge, this is the first study to analyze retinal thickness using OCT by segmenting individual layers in the *APP^NL-F/NL-F^* murine model. As observed when this work was compared with other studies, not all layers exhibited the same behavior over time. Furthermore, segmentation in many previous studies was performed on complexes or grouped layers, potentially masking results and highlighting the need to report values of individual-layer thickness so that the results can be comparable across studies. Finally, the OCT technique enables the analysis of different retinal layer thicknesses in vivo in a specific manner, avoiding potential confusion associated with tissue preparation and fixation [56].

This study highlights the presence of retinal changes at six months of age, marking an early phase in the development of the APP model. During this period, Aβ deposits and dystrophic neurites start to emerge in the cortex and hippocampus [57], although cognitive impairments have not yet surfaced. This model could parallel the natural progression of AD in humans more closely than other transgenic models. Our research, along with numerous clinical studies, indicates that advanced technologies like OCT are highly effective in detecting early retinal changes in AD and could be valuable for disease monitoring [58,59].

## 4. Materials and Methods

### 4.1. Animals Models and Ethics

A total of 72 male mice were used in this study. The WT group consisted of 36 C57BL/6J mice aged 6, 9, 12, 15, 17, and 20 months (n = 6 for each group). The APP^NL/F-NF/L^ group also consisted of 36 mice (n = 6) for each of the ages described above. The APP^NL/F-NF/L^ mouse model is produced by manipulating the mouse APP gene using a knock-in strategy with Swedish (KM670/671NL) and Beyreuther/Iberian (I716F) mutations [35]. The first mutation elevates the total amount of Aβ_40_ and Aβ_42_, while the Beyreuther/Iberian mutation increases the ratio of Aβ_42_/Aβ_40_. This mouse model is bred in homozygosity to accelerate the pathology and eliminate murine endogenous Aβ, and for this reason, the control mice were not littermates.

All animals were housed in the School of Medicine at the University Complutense of Madrid in light- and temperature-controlled rooms with a 12-h light/dark cycle and ad libitum access to food and water.

The project was approved by the Ethics Committee on Animal Welfare of the University Complutense (PROEX No. 047/16) and reported according to the Association for Research in Vision and Ophthalmology (ARVO) statement of animal use. All procedures were performed in accordance with the European Parliament, the Council Directive 2010/63/EU, and Spanish legislation (Real Decreto 53/2013).

### 4.2. Optical Coherence Tomography

The retinas of *APP^NL−F/NL−F^* and WT animals were analyzed using the animal module integrated in the SD-OCT Spectralis with the Heidelberg Eye Explorer software v6.13 (Heidelberg Engineering, Heidelberg, Germany). The procedure followed has been described previously by Salobrar-García et al. [13].

The OCT analysis was conducted while the mice were under general anesthesia using an intraperitoneal injection of a combination of ketamine (75 mg/kg; Anesketin^®^, Dechra Veterinary Products SLU, Barcelona, Spain) and medetomidine (0.26 mg/kg; Medetor^®^, Virbac España S.A., Barcelona, Spain).

During the whole process, the corneas of the mice were moistened with artificial tears, and for pupillary dilation, a drop of tropicamide (tropicamide, 10 mg/mL; colircusi tropicamide, Alcon Healthcare, Barcelona, Spain) was used. The murine eye was covered with a polymethyl methacrylate contact lens (3.2-mm diameter; base curve: 1.7; Cantor&Nissel, Brackley, UK), which served to create a uniform refractive surface. A 25-dioptre mouse lens (Heidelberg, Germany) was added in front of the OCT camera.

For the reversal of anesthesia, a subepithelial injection of atypamezole (Antisedan, 5 mg/mL; Pfizer, NY, USA) was used. After the procedure, the mice were placed in their cages with a heater.

The segmentation of the retinal layer was carried out manually by an expert investigator (L.S-P) using the software Heidelberg Eye v6.13, which was integrated in the SD-OCT Spectralis.

**Figure 5:**
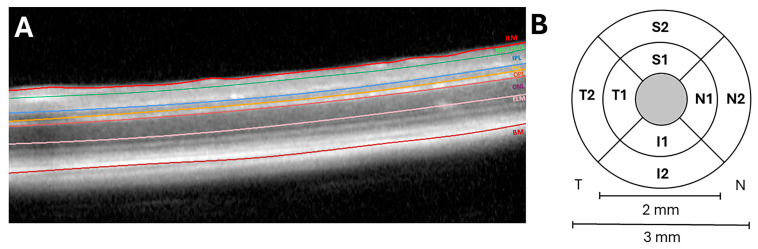
(**A**). Mouse retinal layer observed via optical coherence tomography (OCT). (**B**) Retinal sectors: superior (S), temporal (T), inferior (I), and nasal (N). (1) Sectors of the inner ring (2 mm in diameter) and (2) sectors of the outer ring (3 mm in diameter). The central gray circle has not been analyzed because it corresponds to the exit of the vessels. (ILM: inner limiting membrane; RNFL-GCL: retinal nerve fiber layer and ganglion cell layer complex; IPL: inner plexiform layer; INL: inner nuclear layer; OPL: outer plexiform layer; ONL: outer nuclear layer; ELM: external limiting membrane; and BM: Bruch’s membrane.)

A total of six distinct layers and layer aggregates were considered in the analysis: the retinal nerve fiber layer and the ganglion cell layer complex (RNFL-GCL), the inner plexiform layer (IPL), the inner nuclear layer (INL), the outer plexiform layer (OPL), and the outer nuclear layer (ONL) (Figure 5A).

The results were presented in the Early Treatment Diabetic Retinopathy Study (ETDRS) in three concentric rings with a central area that had a diameter of 1 mm and was not considered for the measurements, an inner ring with a diameter of 2 mm, and an outer ring with a diameter of 3 mm. In addition, each ring was subdivided into four quadrants (superior (S), inferior (I), nasal (N), and temporal (T)) that were named according to (1) the sectors of the inner ring and (2) the sectores of the outer ring (Figure 5B).

The OCT images needed to have a signal-to-noise ratio of at least 25 dB, and they needed to include an average of 16 B-scans to be eligible for inclusion.

### 4.3. Statistical Analysis

GraphPad Prism statistical software, version 9.4.1 (GraphPad Prism, La Jolla, CA, USA), was used for statistical analysis. The data were presented as means ± standard deviations (SDs). The comparison between the WT and *APP^NL-F/NL-F^* groups, with members paired by age, was carried out with the nonparametric Mann–Whitney U test, and for the study, over time, a two-way ANOVA with Tukey correction for multiple comparisons was used. Significance levels were set to * *p* < 0.05, ** *p* < 0.01, and *** *p* < 0.001.

### 4.4. Colorimetric Representation

The representation of thickness changes in each layer or complex layer between the *APP^NL-F/NL-F^* and WT was developed using the colorimetric representation in the ETDRS rings and quadrants. This representation was developed using the Excel software’s version 2406 build 16.0.17726.20078 with color scale function and a normalization of the values. Regions lacking discernible differences are depicted in white. Areas demonstrating thinning in the *APP^NL-F/NL-F^* and WT groups are represented in shades of blue, while those exhibiting thickening appear in shades of red. The color intensity was automatically determined via the software based on the magnitude of the thickness variation.

## 5. Conclusions

In conclusion, both the *APP^NL-F/NL-F^* transgenic model and the C57BL/J6 mouse group exhibited thickness changes at different time points in the study. While the WT mice showed significant changes in INL, OPL, and ONL, the murine AD model showed significant changes in all the retinal layers analyzed.

Furthermore, in this transversal case-control study, the *APP^NL-F/NL-F^* model displayed thickness variations in all retinal layers except the IPL when compared to the age-matched control group. These thickness changes alternated between key thinning and thickening stages, which could be attributed to neurodegenerative and neuroinflammatory processes. These retinal-thickness variations found in the very early stages of this murine model can be used as disease biomarkers, reflecting a determinate stage of the disease. Additionally, these thickness changes closely resemble those found in humans in preclinical stages, as well as in milder and moderate stages of AD, making this murine AD model behave more similarly to the disease in humans.

## Data Availability

Data are available upon request from the corresponding author.

## Supplementary Materials

The following supporting information can be downloaded at https://www.mdpi.com/article/10.3390/ijms25158221/s1.
