# Supplementary material for: Age-Related Retinal Layer Thickness Changes Measured by OCT in APPNL-F/NL-F Mice: Implications for Alzheimer’s Disease
Source: Int J Mol Sci. 2024 Jul 27;25(15):8221. doi: 10.3390/ijms25158221 (PMC11312090; doi:10.3390/ijms25158221)
Supplement: Supplementary file 1 [file ijms-25-08221-s001.zip › Supplementary Table S1.pdf]

Supplementary Table S1. P-Values of the different retinal thickness in each retinal complex or layer between different months of age in the C57Bl6/6J mice. (RNFL: retina nerve fiber layer; GCL: ganglion cell layer; IPL: inner plexiform layer. INL: inner nuclear layer; OPL: outer plexiform layer; ONL: outer nuclear layer. WT: wild type.

Comparison between months/ Retinal sectors	P- Values RNFL+GCL complex							
	N1	N2	S1	S2	T1	T2	I1	I2
6 months vs. 9 months	0.999	0.836	0.797	0.852	0.388	0.217	0.998	0.556
6 months vs. 12 months	>0.9999	0.640	0.992	0.425	0.856	0.987	0.972	0.997
6 months vs. 15 months	0.735	0.698	0.937	0.999	0.759	>0.9999	0.474	>0.9999
6 months vs. 17 months	0.824	0.836	0.854	0.999	0.958	0.900	0.882	0.757
6 months vs. 20 months	0.999	>0.9999	>0.9999	0.413	>0.9999	0.223	0.950	0.828
9 months vs. 12 months	>0.9999	0.945	0.993	0.999	>0.9999	0.987	0.694	0.927
9 months vs. 15 months	0.824	>0.9999	0.658	0.721	>0.9999	0.698	0.601	0.226
9 months vs. 17 months	0.934	>0.9999	1.000	0.791	0.882	0.954	0.934	0.999
9 months vs. 20 months	>0.9999	0.900	0.772	0.398	0.694	1.000	0.754	0.975
12 months vs. 15 months	0.990	0.870	0.854	0.480	>0.9999	0.997	1.000	0.994
12 months vs. 17 months	0.997	0.932	0.999	0.640	0.900	>0.9999	0.326	0.932
12 months vs. 20 months	>0.9999	0.616	0.961	0.088	0.856	0.997	>0.9999	0.966
15 months vs. 17 months	0.999	>0.9999	0.693	>0.9999	0.951	0.980	0.248	0.548
15 months vs. 20 months	0.754	0.358	0.824	0.900	0.652	0.828	>0.9999	0.560
17 months vs. 20 months	0.980	0.836	0.854	0.932	0.978	0.987	0.370	0.958
**Comparison between months/ Retinal sectors**	**P- Values IPL**							
	**N1**	**N2**	**S1**	**S2**	**T1**	**T2**	**I1**	**I2**
6 months vs. 9 months	>0.9999	0.900	0.996	0.867	0.999	>0.9999	0.993	0.981
6 months vs. 12 months	0.949	0.529	0.980	0.427	>0.9999	0.987	0.962	0.997
6 months vs. 15 months	0.980	0.663	0.807	0.998	0.184	0.398	>0.9999	0.850
6 months vs. 17 months	0.999	0.617	0.883	0.997	>0.9999	0.999	0.843	0.849
6 months vs. 20 months	>0.9999	0.987	0.982	0.156	0.837	0.869	0.997	0.973
9 months vs. 12 months	0.931	>0.9999	0.944	0.999	0.997	0.941	0.836	>0.9999

9 months vs. 15 months	0.985	>0.9999	>0.9999	0.851	0.588	0.463	0.999	0.975
9 months vs. 17 months	0.995	0.941	0.709	0.398	>0.9999	0.994	0.998	0.298
9 months vs. 20 months	>0.9999	0.491	0.966	0.320	0.847	0.995	0.967	>0.9999
12 months vs. 15 months	>0.9999	0.999	0.849	0.809	0.487	0.895	0.983	0.988
12 months vs. 17 months	0.640	0.927	0.999	0.650	0.999	0.990	0.503	0.417
12 months vs. 20 months	0.900	0.432	0.999	0.074	0.996	0.871	0.999	>0.9999
15 months vs. 17 months	0.936	0.974	0.592	0.417	0.544	0.122	0.997	0.999
15 months vs. 20 months	0.994	0.721	0.698	0.184	0.242	0.349	0.997	0.988
17 months vs. 20 months	0.992	0.398	0.979	0.966	0.949	0.969	0.919	0.978
**Comparison between months/ Retinal sectors**	**P- Values INL**							
	**N1**	**N2**	**S1**	**S2**	**T1**	**T2**	**I1**	**I2**
6 months vs. 9 months	0.234	0.999	0.631	0.546	0.567	0.811	0.640	>0.9999
6 months vs. 12 months	0.997	0.991	0.936	0.934	>0.9999	0.859	0.940	0.970
6 months vs. 15 months	0.999	0.999	0.856	0.985	0.999	0.785	0.982	>0.9999
6 months vs. 17 months	0.072	**0.010**	**0.020**	0.915	**0.003**	0.180	0.095	0.064
6 months vs. 20 months	0.997	0.474	0.951	0.996	**0.018**	0.662	0.290	0.704
9 months vs. 12 months	0.694	0.954	0.933	0.984	0.915	>0.9999	0.499	0.937
9 months vs. 15 months	0.601	0.991	0.954	0.970	0.992	>0.9999	0.567	>0.9999
9 months vs. 17 months	**0.043**	0.102	0.253	0.497	**0.008**	**0.001**	**0.045**	**0.032**
9 months vs. 20 months	0.473	0.694	0.710	0.712	0.067	0.159	0.168	0.601
12 months vs. 15 months	0.999	0.999	0.999	0.958	0.980	0.991	>0.9999	0.142
12 months vs. 17 months	0.601	0.698	0.058	0.529	0.315	0.131	0.531	0.606
12 months vs. 20 months	0.869	0.937	0.640	0.940	0.186	0.494	0.961	0.994
15 months vs. 17 months	0.472	0.602	0.064	0.663	0.200	0.090	0.725	0.288
15 months vs. 20 months	0.997	0.956	0.738	0.966	0.142	0.499	0.993	0.886
17 months vs. 20 months	0.964	0.991	0.601	0.990	0.958	0.388	0.217	0.447
	**P- Values OPL**							

Comparison between months/ Retinal sectors	N1	N2	S1	S2	T1	T2	I1	I2
6 months vs. 9 months	0.064	0.064	0.602	0.966	0.218	0.122	0.184	0.296
6 months vs. 12 months	>0.9999	0.869	0.735	0.828	0.560	0.698	0.640	0.958
6 months vs. 15 months	0.859	0.891	0.358	0.180	0.991	0.991	0.824	0.836
6 months vs. 17 months	0.978	0.824	0.754	0.326	>0.9999	0.922	0.975	0.999
6 months vs. 20 months	0.759	0.326	0.999	0.759	>0.9999	>0.9999	0.987	0.975
9 months vs. 12 months	0.226	0.142	0.217	0.754	0.226	0.276	0.180	0.417
9 months vs. 15 months	0.280	0.270	0.184	0.175	0.077	0.180	0.103	0.103
9 months vs. 17 months	0.217	0.247	0.290	0.218	**0.020**	**0.013**	**0.022**	0.307
9 months vs. 20 months	0.082	0.058	0.809	0.662	0.217	0.092	**0.014**	**0.045**
12 months vs. 15 months	0.560	0.980	0.824	0.077	0.072	0.991	0.307	0.560
12 months vs. 17 months	0.900	0.978	0.999	0.662	0.223	0.388	0.291	0.997
12 months vs. 20 months	0.472	0.567	0.958	0.987	0.754	0.934	0.217	0.662
15 months vs. 17 months	>0.9999	>0.9999	0.223	0.991	0.999	0.900	0.999	0.869
15 months vs. 20 months	0.999	0.997	0.640	0.828	0.994	>0.9999	0.994	>0.9999
17 months vs. 20 months	0.836	0.991	0.966	0.975	0.999	0.869	0.997	0.869

Comparison between months/ Retinal sectors	P- Values ONL							
	N1	N2	S1	S2	T1	T2	I1	I2
6 months vs. 9 months	0.625	0.824	0.566	0.999	0.982	0.313	0.797	0.986
6 months vs. 12 months	0.999	0.946	0.364	0.934	0.587	0.991	0.993	>0.9999
6 months vs. 15 months	0.451	0.764	0.698	0.395	0.061	**0.034**	0.723	0.630
6 months vs. 17 months	0.411	0.993	0.943	0.997	**0.003**	0.528	0.535	0.884
6 months vs. 20 months	0.257	0.088	0.226	0.056	0.574	**0.034**	0.238	**0.032**
9 months vs. 12 months	0.783	0.999	0.762	>0.9999	0.972	0.978	0.705	0.970
9 months vs. 15 months	0.139	0.372	0.195	0.293	0.306	0.180	0.101	0.991
9 months vs. 17 months	0.172	0.669	0.127	0.937	0.304	0.913	0.192	0.999

9 months vs. 20 months	0.131	0.082	0.053	0.143	0.821	0.115	0.146	0.690
12 months vs. 15 months	0.336	0.183	**0.048**	**0.013**	0.411	0.168	0.843	0.506
12 months vs. 17 months	0.133	0.311	0.467	0.816	0.412	0.435	0.204	0.350
12 months vs. 20 months	0.238	0.059	0.084	**0.003**	>0.9999	**0.044**	0.526	0.156
15 months vs. 17 months	>0.9999	0.997	0.999	0.908	>0.9999	0.978	0.943	0.999
15 months vs. 20 months	0.974	0.071	0.599	0.411	0.277	0.966	0.853	0.313
17 months vs. 20 months	0.999	0.527	0.129	0.332	0.468	0.894	>0.9999	0.640
