# Supplementary material for: Age-Related Retinal Layer Thickness Changes Measured by OCT in APPNL-F/NL-F Mice: Implications for Alzheimer’s Disease
Source: Int J Mol Sci. 2024 Jul 27;25(15):8221. doi: 10.3390/ijms25158221 (PMC11312090; doi:10.3390/ijms25158221)
Supplement: Supplementary file 1 [file ijms-25-08221-s001.zip › Supplementary Table S2.pdf]

Supplementary Table S2. P-Values of the different retinal thickness in each retinal complex or layer between different months of age in the APP $^{NL-F/NL-F}$ mouse model. (RNFL: retina nerve fiber layer; GCL: ganglion cell layer; IPL: inner plexiform layer. INL: inner nuclear layer; OPL: outer plexiform layer; ONL: outer nuclear layer. WT: wild type.

Comparison between months/ Retinal sectors	P- Values RNFL+GCL complex							
	**N1**	**N2**	**S1**	**S2**	**T1**	**T2**	**I1**	**I2**
6 months vs. 9 months	0.997	0.958	0.964	0.995	0.964	0.869	0.159	>0.9999
6 months vs. 12 months	>0.9999	0.997	0.999	0.911	>0.9999	0.980	0.662	0.900
6 months vs. 15 months	0.975	0.640	0.531	0.735	0.276	0.142	0.142	0.288
6 months vs. 17 months	0.704	0.979	0.974	0.547	0.388	0.662	**0.034**	0.074
6 months vs. 20 months	0.826	0.900	>0.9999	0.882	0.882	0.662	0.652	0.393
9 months vs. 12 months	0.991	0.994	0.995	0.965	0.869	>0.9999	0.836	0.951
9 months vs. 15 months	>0.9999	0.999	0.991	0.995	0.940	0.523	0.974	0.432
9 months vs. 17 months	0.999	>0.9999	>0.9999	0.455	0.990	0,999	0.975	0.331
9 months vs. 20 months	0.999	0.606	0.927	0.754	>0.9999	0.999	0.937	0.092
12 months vs. 15 months	0.987	0.998	0.529	0.590	0.494	0.122	0.370	0.290
12 months vs. 17 months	0.908	0.994	0.995	0.814	0.472	0.999	0.548	0.927
12 months vs. 20 months	0.474	0.447	>0.9999	0.996	0.828	0.991	0.998	0.900
15 months vs. 17 months	0.915	>0.9999	0.857	0.120	0.980	0.604	0.999	0.985

15 months vs. 20 months	0.754	0.683	0.407	0.250	0.735	0.441	0.900	0.999
17 months vs. 20 months	>0.9999	0.331	0.869	0.980	0.999	>0.9999	0.843	0.994

**Comparison between months/ Retinal sectors**	**P- Values IPL**							
	**N1**	**N2**	**S1**	**S2**	**T1**	**T2**	**I1**	**I2**
6 months vs. 9 months	0.952	>0.9999	0.923	0.834	0,999	0.934	0.857	0.922
6 months vs. 12 months	0.873	>0.9999	0.991	0.956	>0.9999	0.409	0.922	0.078
6 months vs. 15 months	0.367	0.983	0.640	0.491	0.647	0.127	0.217	0.223
6 months vs. 17 months	0.878	>0.9999	0.671	0.982	0.719	0.326	0.602	0.112
6 months vs. 20 months	0.126	0.148	**0.022**	0.280	**0.014**	0.092	0.102	**0.023**
9 months vs. 12 months	0.997	>0.9999	0.956	0.917	0.999	0.999	0.904	0.999
9 months vs. 15 months	0,999	>0.9999	>0.9999	0.996	0.775	0.634	>0.9999	>0.9999
9 months vs. 17 months	0,999	>0.9999	>0.9999	0.761	0.814	0.932	0.976	0.998
9 months vs. 20 months	0.839	0.936	0.537	>0.9999	0.721	0.764	>0.9999	0.894
12 months vs. 15 months	0.759	0.978	0.983	0.964	0.326	0.218	**0.010**	0.996
12 months vs. 17 months	0,999	>0.9999	0.954	>0.9999	0.326	0.987	0.991	>0.9999
12 months vs. 20 months	**0.022**	0.313	0.330	0.589	0.322	0.345	0.560	0.367

15 months vs. 17 months	0.974	0.978	>0.9999	0.870	>0.9999	0.223	0.927	0.997
15 months vs. 20 months	0.560	0.703	**0.030**	0.836	0.739	0.991	0.998	0.502
17 months vs. 20 months	0.502	0.386	0.178	0.343	0.647	0.599	0.891	0.669

**Comparison between months/ Retinal sectors**	**P- Values INL**							
	**N1**	**N2**	**S1**	**S2**	**T1**	**T2**	**I1**	**I2**
6 months vs. 9 months	0,999	0.980	0.996	0.800	0.995	0.944	0.998	0.857
6 months vs. 12 months	0.825	0.567	0.509	0.247	0.683	0.265	0.601	0.095
6 months vs. 15 months	0.503	0.186	**0.022**	0.118	0.072	0.163	**0.033**	0.082
6 months vs. 17 months	0.472	0.115	0.486	0.183	0.393	0.118	0.217	**0.002**
6 months vs. 20 months	0.168	0.211	0.175	0.249	0.291	0.142	0.473	0.118
9 months vs. 12 months	0.950	0.911	0.837	0.991	0.920	0.836	0.828	0.529
9 months vs. 15 months	0.954	0.725	0.560	0.910	0.588	0.738	0.199	0.814
9 months vs. 17 months	0.531	0.247	0.839	0.918	0.788	0.543	0.413	0.226
9 months vs. 20 months	0.432	0.674	0.711	0.996	0.683	0.711	0.793	0.389
12 months vs. 15 months	0.999	0.927	0.951	0.995	0.601	0.997	0.067	0,999
12 months vs. 17 months	0.735	0.169	>0.9999	0.991	0.990	0.735	0.754	0.567

12 months vs. 20 months	0.307	0.987	0,999	0.999	0.824	0.958	0.980	0.994
15 months vs. 17 months	0.980	0.789	0.988	0,999	0.735	0.698	0.072	0.291
15 months vs. 20 months	0.698	0.966	0.869	0.811	0.900	>0.9999	0.472	0.980
17 months vs. 20 months	0.836	0.381	>0.9999	0.729	0.998	0.358	0,999	0.964

**Comparison between months/ Retinal sectors**	**P- Values OPL**							
	**N1**	**N2**	**S1**	**S2**	**T1**	**T2**	**I1**	**I2**
6 months vs. 9 months	0.159	0.671	0.413	0.996	0.407	0.694	0.589	0.735
6 months vs. 12 months	0.159	0.462	0.407	0.290	0.276	0.234	0.494	0.704
6 months vs. 15 months	**0.042**	0.142	**0.006**	0.389	0.054	0.064	0.494	0.859
6 months vs. 17 months	0.226	0.546	0.115	0.358	0.969	0.991	0.987	0.936
6 months vs. 20 months	0.462	**0.039**	0.293	0.407	0.893	0.878	0.548	0,999
9 months vs. 12 months	>0.9999	0.998	0,999	0.674	>0.9999	0.999	>0.9999	0.996
9 months vs. 15 months	>0.9999	0.987	0.731	0.326	0.998	>0.9999	>0.9999	0.936
9 months vs. 17 months	0.984	>0.9999	0.999	0.729	0.971	0.990	0.915	0.223
9 months vs. 20 months	0,999	>0.9999	0.969	0.802	0,999	>0.9999	>0.9999	0.877
12 months vs. 15 months	0.999	0.291	0.744	>0.9999	0.970	0.999	>0.9999	0.698

12 months vs. 17 months	0.934	0.966	>0.9999	0.998	0.974	0.940	0.807	**0.020**
12 months vs. 20 months	0.999	0.974	0.987	>0.9999	0,999	>0.9999	>0.9999	0.759
15 months vs. 17 months	0.447	0.922	0.811	0.997	0.719	0.956	0.602	0.067
15 months vs. 20 months	0.987	0.997	0.999	>0.9999	0.888	>0.9999	>0.9999	0.824
17 months vs. 20 months	0.999	>0.9999	0.978	0,999	0.999	0.983	0.836	0.873

**Comparison between months/ Retinal sectors**	**P- Values ONL**							
	**N1**	**N2**	**S1**	**S2**	**T1**	**T2**	**I1**	**I2**
6 months vs. 9 months	0.168	0.118	**0.025**	**0.020**	**0.025**	**0.002**	0.147	**0.033**
6 months vs. 12 months	0.623	0.672	0.419	>0.9999	0.288	0.538	0.836	0.180
6 months vs. 15 months	0.757	0.361	0.979	>0.9999	**0.024**	0.217	>0.9999	0.704
6 months vs. 17 months	0.684	0.625	0.995	0.738	0.445	0.325	0.602	0.131
6 months vs. 20 months	>0.9999	>0.9999	0.463	**0.018**	>0.9999	0.977	0.074	0.997
9 months vs. 12 months	0.467	0.239	0.276	0.103	0.457	**0.024**	0.270	0.643
9 months vs. 15 months	0.187	**0.049**	0.058	**0.013**	0.509	**0.013**	0.338	**0.016**
9 months vs. 17 months	0.399	0.151	**0.018**	**0.015**	0.314	**0.017**	0.673	0.103
9 months vs. 20 months	**0.032**	0.128	**0.002**	**0.001**	0.087	**0.028**	0.055	**0.011**

12 months vs. 15 months	0.999	0.962	0.971	0.999	0.945	0.986	0.979	0.943
12 months vs. 17 months	>0.9999	0.999	0.869	0.358	>0.9999	0.946	>0.9999	0.729
12 months vs. 20 months	0.724	0.339	0.190	0.232	0.667	0.978	0.186	0.301
15 months vs. 17 months	0.956	0.972	0.999	0.494	0.964	0.999	0.945	>0.9999
15 months vs. 20 months	0.719	0.485	0.424	0.069	0.556	0.601	0.763	0.640
17 months vs. 20 months	0.560	0.175	0.210	0.548	0.580	0.372	0.167	0.175
