# Supplementary material for: Age-Related Retinal Layer Thickness Changes Measured by OCT in APPNL-F/NL-F Mice: Implications for Alzheimer’s Disease
Source: Int J Mol Sci. 2024 Jul 27;25(15):8221. doi: 10.3390/ijms25158221 (PMC11312090; doi:10.3390/ijms25158221)
Supplement: Supplementary file 1 [file ijms-25-08221-s001.zip › Supplementary Table S3.pdf]

**Supplementary Table S3.** Table 1. Retinal thickness in each retinal complex or layer at different months of age for the two study groups. The values are expressed as mean ± standard deviation. (RNFL: retina nerve fiber layer; GCL: ganglion cell layer; IPL: inner plexiform layer, INL: inner nuclear layer; OPL: outer plexiform layer; ONL: outer nuclear layer, WT: wild type.

| | **Retinal complex/ layers** | **RNFL+GCL** | | | **IPL** | | | **INL** | | | **OPL** | | | **ONL** | | |
|---|---|---|---|---|---|---|---|---|---|---|---|---|---|---|---|---|
| **Months of age** | **Retinal sectors** | **WT** MEAN±SD | **$APP^{NL-F/NL-F}$** MEAN±SD | **P-Value** | **WT** MEAN±SD | **$APP^{NL-F/NL-F}$** MEAN±SD | **P-Value** | **WT** MEAN±SD | **$APP^{NL-F/NL-F}$** MEAN±SD | **P-Value** | **WT** MEAN±SD | **$APP^{NL-F/NL-F}$** MEAN±SD | **P-Value** | **WT** MEAN±SD | **$APP^{NL-F/NL-F}$** MEAN±SD | **P-Value** |
| **6 Months** | **N1** | 23.33±1.033 | 20.67±2.251 | **0.043** | 46.17±3.125 | 47.5±1.378 | 0.323 | 19.5±0.5477 | 18.67±1.751 | 0.708 | 19.83±1.602 | 20.67±1.033 | 0.446 | 51.33±4.633 | 43.33±2.733 | **0.011** |
| | **N2** | 21.17±0.7528 | 20.83±1.472 | >0.999999 | 47.33±2.944 | 46.17±3.312 | 0.615 | 20.33±0.5164 | 18.33±1.633 | 0.032 | 19.5±1.643 | 21±1.789 | 0.167 | 48.83±5.154 | 41±3.033 | **0.022** |
| | **S1** | 24.67±1.751 | 23.33±1.366 | 0.199 | 45.33±3.011 | 47.5±3.017 | 0.301 | 20.67±1.033 | 19±1.549 | 0.067 | 19.67±1.033 | 22±0.6325 | **0.004** | 47.33±3.983 | 43.5±3.564 | 0.095 |
| | **S2** | 22±2.098 | 23.17±2.787 | 0.377 | 46.83±2.858 | 47±4 | 0.623 | 20.5±1.643 | 18.67±1.211 | 0.097 | 19.83±1.472 | 22±0.8944 | **0.013** | 50.5±3.728 | 46.83±3.488 | 0.206 |
| | **T1** | 22.5±1.049 | 21.33±0.8165 | 0.108 | 47.17±3.371 | 46.33±2.16 | 0.719 | 20.33±0.8165 | 18±1.265 | 0.009 | 20.17±1.329 | 20.67±1.033 | 0.652 | 55.33±3.983 | 42.5±1.643 | **0.002** |
| | **T2** | 21.67±0.5164 | 20.17±1.472 | 0.080 | 47±2.608 | 48.33±3.077 | 0.673 | 20.67±1.211 | 18.67±0.8165 | 0.022 | 21±1.095 | 21.67±0.8165 | 0.489 | 52±3.347 | 40.17±3.061 | **0.002** |
| | **I1** | 23.67±1.633 | 21.33±0.5164 | **0.013** | 46.17±2.401 | 46.5±1.761 | 0.779 | 21.33±1.862 | 19.17±1.329 | 0.078 | 21±1.265 | 20.83±0.9832 | 0.777 | 51±4.336 | 44.33±0.8165 | **0.009** |
| | **I2** | 22.67±1.506 | 19.67±1.211 | **0.013** | 47.83±4.579 | 48.5±2.168 | 0.861 | 21.5±0.5477 | 18.5±0.5477 | 0.002 | 22.17±1.169 | 21.33±1.211 | 0.355 | 53.5±4.764 | 44.5±2.429 | **0.004** |
| **9 Months** | **N1** | 23.17±1.169 | 21.5±2.811 | 0.182 | 46±2.28 | 45.83±4.446 | 0.853 | 21±1.265 | 19.17±3.189 | 0.517 | 18.5±1.225 | 18.83±0.9832 | 0.864 | 57.33±8.287 | 48.67±1.966 | **0.019** |
| | **N2** | 21.67±0.8165 | 19.83±2.229 | 0.113 | 45.5±2.168 | 45.67±5.279 | 0.519 | 20.5±1.049 | 19.33±2.733 | 0.483 | 18.17±1.472 | 19±2.191 | 0.491 | 52.33±6.89 | 48.67±2.733 | 0.461 |
| | **S1** | 26.67±3.204 | 24.33±2.582 | 0.264 | 44±4.94 | 45.67±2.658 | 0.413 | 23.33±4.367 | 19.67±3.011 | 0.139 | 18.5±1.871 | 20.5±1.517 | 0.091 | 56±9.274 | 48.83±1.941 | 0.251 |

| | | | | | | | | | | | | | | | | |
|---|---|---|---|---|---|---|---|---|---|---|---|---|---|---|---|---|
| | **S2** | 24±3.098 | 24.17±4.07 | 0.987 | 44.33±3.204 | 43.17±5.037 | 0.619 | 22.5±3.271 | 20.33±3.011 | 0.212 | 19.33±2.16 | 21.67±1.033 | 0.056 | 51.67±5.203 | 51.17±2.317 | 0.903 |
| | **T1** | 23.67±0.5164 | 22±1.414 | **0.045** | 46.5±1.761 | 47±4.29 | 0.656 | 21.33±1.211 | 18.67±2.658 | 0.113 | 18±1.549 | 18.83±1.941 | 0.446 | 53.67±6.186 | 49.17±1.835 | 0.160 |
| | **T2** | 20.67±0.8165 | 20.83±0.7528 | 0.892 | 47.17±2.137 | 46.17±4.07 | 0.851 | 21.67±1.033 | 19.5±2.51 | 0.145 | 18.83±1.169 | 20.5±1.871 | 0.147 | 49.17±3.371 | 49.33±1.633 | 0.911 |
| | **I1** | 23.33±1.211 | 23.17±1.169 | 0.935 | 45.33±1.966 | 44.17±3.971 | 0.790 | 22.33±1.211 | 19.67±1.862 | 0.035 | 18.33±1.366 | 19.17±1.835 | 0.584 | 53±2.828 | 47.83±2.229 | **0.011** |
| | **I2** | 21.33±1.033 | 19.67±1.506 | 0.084 | 46.5±2.074 | 46.33±4.179 | 0.950 | 21.5±0.8367 | 19.67±2.066 | 0.171 | 19.33±1.862 | 19.67±1.966 | 0.745 | 51.33±6.121 | 50.67±1.033 | 0.848 |
| **12 Months** | **N1** | 23.33±3.445 | 20.83±1.472 | 0.104 | 47.5±2.665 | 46.5±1.225 | 0.524 | 19.83±1.722 | 20.17±1.722 | 0.864 | 19.83±0.7528 | 18.83±1.722 | 0.431 | 51.83±4.215 | 45.33±3.141 | **0.022** |
| | **N2** | 22.83±2.787 | 20.5±2.168 | 0.128 | 45.17±3.189 | 46±1.414 | 0.794 | 20±1.673 | 20.33±1.366 | 0.788 | 20.17±1.169 | 19.5±1.643 | 0.552 | 51.33±4.803 | 43.5±3.834 | **0.019** |
| | **S1** | 25.5±3.017 | 23.5±2.345 | 0.260 | 47±6.293 | 46.5±1.871 | 0.619 | 21.5±1.643 | 21.33±1.862 | 0.905 | 20.5±1.049 | 20.17±2.483 | 0.998 | 50±4.05 | 45.5±3.082 | 0.076 |
| | **S2** | 25±4.147 | 22.17±3.125 | 0.403 | 43.5±4.889 | 45.5±2.51 | 0.626 | 21.5±2.074 | 21.17±1.941 | >0.999999 | 20.67±0.5164 | 20.17±2.137 | 0.805 | 51.83±0.7528 | 47±3.406 | **0.006** |
| | **T1** | 23.5±2.258 | 21.33±1.033 | **0.039** | 47.33±3.83 | 46.5±1.378 | 0.848 | 20.17±2.401 | 19.67±1.751 | 0.894 | 19.33±0.5164 | 19±2.191 | >0.999999 | 51.33±4.457 | 45.83±2.858 | **0.045** |
| | **T2** | 21.17±1.835 | 20.67±1.862 | 0.571 | 46±3.521 | 45.5±1.517 | 0.810 | 21.5±2.074 | 21±1.673 | 0.751 | 20.5±0.8367 | 20.17±1.602 | 0.784 | 50.67±3.933 | 43.17±3.656 | **0.011** |
| | **I1** | 24.5±1.871 | 22.17±1.169 | **0.022** | 47.83±4.834 | 45.83±0.9832 | 0.868 | 20.33±1.966 | 20.5±1.049 | 0.838 | 20.33±1.033 | 19.17±1.722 | 0.184 | 50.17±4.167 | 45.33±1.966 | **0.028** |
| | **I2** | 22.33±1.966 | 20.5±2.074 | 0.206 | 46.67±3.559 | 45.67±1.033 | 0.552 | 20.83±1.941 | 21.33±1.633 | 0.506 | 21.83±1.169 | 20±1.265 | **0.048** | 53.67±4.367 | 48.5±2.881 | **0.037** |
| **15 Months** | **N1** | 22.5±0.5477 | 21.17±1.472 | 0.130 | 47.17±3.061 | 45.33±1.633 | 0.333 | 19.67±1.751 | 20.5±1.517 | 0.489 | 20.67±1.862 | 18.67±1.366 | 0.113 | 46.5±2.258 | 44.83±1.602 | 0.188 |
| | **N2** | 21.67±0.8165 | 20.17±1.169 | **0.043** | 45.5±3.619 | 45.33±2.066 | 0.654 | 20.17±1.722 | 21.17±1.472 | 0.340 | 20.67±2.251 | 18.33±1.033 | 0.067 | 45.5±2.258 | 44.67±1.862 | 0.476 |
| | **S1** | 24±1.265 | 25.17±2.401 | 0.255 | 44.17±1.169 | 45.5±2.258 | 0.355 | 21.67±1.506 | 22.17±1.169 | 0.481 | 21.17±0.9832 | 19.17±1.472 | **0.035** | 44.83±2.858 | 44.5±1.975 | 0.814 |
| | **S2** | 21.5±1.871 | 24.83±2.858 | **0.024** | 46.17±1.835 | 44.17±2.714 | 0.210 | 21.17±2.041 | 21.83±1.941 | 0.565 | 21.83±0.7528 | 20±1.789 | 0.067 | 46.67±2.582 | 46.67±1.633 | 0.810 |

| | | | | | | | | | | | | | | | | |
|---|---|---|---|---|---|---|---|---|---|---|---|---|---|---|---|---|
| | **T1** | 23.67±1.366 | 23±1.789 | 0.446 | 44.17±1.722 | 44.83±1.169 | 0.565 | 20.67±2.066 | 21±1.095 | 0.680 | 20.33±0.8165 | 18.33±1.366 | **0.022** | 46.67±1.966 | 46.83±2.137 | 0.981 |
| | **T2** | 21.67±1.366 | 22.83±2.483 | 0.483 | 44.5±1.517 | 43.33±1.633 | 0.216 | 21.83±1.941 | 21.33±1.506 | 0.825 | 20.83±1.472 | 20.33±1.211 | 0.450 | 45±2.098 | 44.17±1.941 | 0.567 |
| | **I1** | 24.67±1.366 | 24±2.28 | 0.418 | 45.83±4.535 | 44.5±0.8367 | 0.736 | 20.33±2.066 | 22.17±0.7528 | 0.093 | 21.67±1.366 | 19.17±1.941 | **0.039** | 47.67±1.633 | 44.5±2.258 | **0.026** |
| | **I2** | 22.67±0.5164 | 22.33±2.658 | 0.385 | 45.5±2.258 | 46±2.098 | 0.777 | 21.5±1.517 | 21.17±1.722 | 0.946 | 22.67±1.033 | 20.5±1.049 | **0.015** | 49.83±1.472 | 47.17±2.137 | **0.041** |
| **17 Months** | **N1** | 22.67±0.8165 | 22±1.897 | 0.621 | 45.5±3.017 | 46.17±2.927 | 0.690 | 18.5±0.5477 | 21±1.414 | 0.009 | 20.5±1.049 | 19.33±1.506 | 0.234 | 46.67±3.983 | 45.67±2.251 | 0.294 |
| | **N2** | 21.67±1.033 | 20±2.28 | 0.119 | 44.33±3.204 | 46±1.095 | 0.381 | 19±0 | 22±1.265 | 0.002 | 20.83±1.472 | 19±2.449 | 0.180 | 46.67±6.501 | 43.83±2.994 | 0.115 |
| | **S1** | 26.17±3.545 | 24±1.673 | 0.374 | 48.33±5.574 | 45.5±1.871 | 0.251 | 18.5±0.5477 | 21.5±2.258 | 0.028 | 20.33±0.5164 | 20.17±1.472 | >0.999999 | 45.33±2.875 | 44.17±1.169 | 0.171 |
| | **S2** | 21.67±2.805 | 20.67±2.066 | 0.905 | 47.83±3.312 | 45.67±2.338 | 0.258 | 19.33±1.751 | 22±2.098 | 0.039 | 21.5±1.049 | 20.5±1.643 | 0.374 | 49.33±4.502 | 45±1.673 | **0.024** |
| | **T1** | 22.83±1.722 | 22.5±1.225 | 0.803 | 46.83±3.312 | 44.83±0.9832 | 0.258 | 17.67±0.8165 | 20.17±1.472 | 0.015 | 20.17±0.9832 | 19.83±2.137 | 0.866 | 47±3.225 | 45.67±2.875 | 0.212 |
| | **T2** | 21.17±1.329 | 21±1.414 | 0.909 | 46.67±1.211 | 45±1.095 | 0.067 | 18.67±0.8165 | 21.83±1.602 | 0.006 | 21.67±1.211 | 21.17±1.722 | 0.654 | 46.83±4.997 | 44.5±2.811 | 0.095 |
| | **I1** | 22.83±0.7528 | 23.67±1.366 | 0.314 | 45±1.414 | 45.33±2.16 | 0.892 | 18.5±1.517 | 21.17±0.7528 | 0.011 | 21.5±0.8367 | 20.33±1.966 | 0.184 | 45.67±5.164 | 45.5±1.761 | 0.699 |
| | **I2** | 21.17±1.835 | 21.5±0.8367 | 0.784 | 45±3.347 | 45.67±1.033 | 0.788 | 18.83±1.472 | 22.33±0.8165 | 0.002 | 22±1.095 | 22.17±1.329 | 0.987 | 50.33±3.983 | 47.33±1.506 | 0.158 |
| **20 Months** | **N1** | 23.17±1.169 | 21.83±1.329 | 0.143 | 46.33±2.338 | 43.67±2.16 | 0.082 | 19.17±1.722 | 21.5±0.8367 | 0.032 | 21±0.8944 | 19.17±2.787 | 0.253 | 47.17±1.941 | 43.17±2.137 | **0.009** |
| | **N2** | 21.17±0.7528 | 21.83±2.137 | 0.537 | 47.83±2.229 | 43±2.828 | **0.006** | 19.33±1.506 | 20.67±1.033 | 0.156 | 21.17±0.7528 | 18.83±2.858 | 0.076 | 40.83±2.137 | 41.33±4.179 | 0.857 |
| | **S1** | 24.67±1.033 | 23.33±0.8165 | 0.065 | 46.17±3.189 | 42.33±2.582 | 0.056 | 19.83±2.401 | 21.5±0.5477 | 0.141 | 19.83±1.329 | 19.5±2.588 | >0.999999 | 42±2.28 | 40.83±3.43 | 0.671 |
| | **S2** | 20.5±1.225 | 21.5±1.871 | 0.409 | 49.17±2.041 | 42.67±2.582 | **0.002** | 20.17±2.563 | 20.83±0.9832 | 0.578 | 21±1.265 | 20.17±2.483 | 0.561 | 43.5±2.881 | 42.83±2.401 | 0.597 |
| | **T1** | 22.5±1.761 | 22.17±1.722 | 0.745 | 48.5±3.782 | 43.33±2.16 | **0.032** | 18±1.265 | 20.33±1.506 | 0.011 | 20±0.8944 | 19.33±2.875 | >0.999999 | 51.17±4.309 | 42.83±4.167 | **0.015** |

| | | | | | | | | | | | | | | | |
|---|---|---|---|---|---|---|---|---|---|---|---|---|---|---|---|
| | **T2** | 20.83±0.7528 | 21±0.6325 | 0.864 | 47.67±2.805 | 42.83±2.639 | **0.006** | 19.83±1.329 | 21.33±1.366 | 0.080 | 21±0.8944 | 20.33±2.805 | >0.999999 | 44.5±2.074 | 41.5±4.889 | 0.335 |
| | **I1** | 24.67±1.862 | 22.5±1.517 | 0.097 | 47.17±4.622 | 44.17±2.137 | 0.234 | 19.5±2.074 | 21±1.789 | 0.247 | 21.33±0.5164 | 19.33±2.805 | 0.385 | 45.5±3.937 | 42.5±1.871 | 0.203 |
| | **I2** | 21.83±1.169 | 21.83±1.472 | 0.989 | 46.5±2.881 | 43.5±3.209 | 0.177 | 20.17±2.229 | 21.67±2.066 | 0.420 | 22.67±0.8165 | 21.17±1.941 | 0.201 | 47±2.683 | 44.83±1.941 | 0.167 |
